# Harnessing Neuroimaging to Reduce Socioeconomic Disparities in Chronic Disease: A Conceptual Framework for Improving Health Messaging

**DOI:** 10.3389/fnhum.2021.576749

**Published:** 2021-02-02

**Authors:** Samantha N. Brosso, Paschal Sheeran, Allison J. Lazard, Keely A. Muscatell

**Affiliations:** ^1^Department of Psychology and Neuroscience, University of North Carolina at Chapel Hill, Chapel Hill, NC, United States; ^2^Lineberger Comprehensive Cancer Center, University of North Carolina at Chapel Hill, Chapel Hill, NC, United States; ^3^Hussman School of Journalism and Media, University of North Carolina at Chapel Hill, Chapel Hill, NC, United States; ^4^Carolina Population Center, University of North Carolina at Chapel Hill, Chapel Hill, NC, United States

**Keywords:** social neuroscience, communication neuroscience, predictive brain, socioeconomic health disparities, disease prevention, health messaging, health behavior change

## Abstract

Socioeconomic status (SES)-related health disparities persist for numerous chronic diseases, with lower-SES individuals exhibiting greater risk of morbidity and mortality compared to their higher-SES counterparts. One likely contributor is disparities in health messaging efforts, which are currently less effective for motivating health behavior change among those lower in SES. Drawing on communication neuroscience and social neuroscience research, we describe a conceptual framework to improve health messaging effectiveness in lower SES communities. The framework is based on evidence that health-message-induced activity in the ventral striatum (VS) and subdivisions of the medial pre-frontal cortex (MPFC) predicts behavior change. Additionally, we draw from social neuroscience work showing that activity in these regions during valuation and the processing of self-related vs. social information, differs as a function of SES. Bringing together these previously disparate lines of work, we argue that health messages emphasizing the *benefits to close others* (vs. the self) of engaging in behavior change will be more effective among lower SES individuals. We also outline a research agenda based on our framework. Ultimately, we hope that this framework utilizing a “brain-as-predictor” approach generates novel insights about the neural underpinnings of message-induced behavior change among lower SES individuals, and helps to close the gap in SES-based health disparities by harnessing the power of neuroimaging.

## Introduction

### Socioeconomic-Based Disparities in Health and Health Communication

Socioeconomic-based disparities in health outcomes are well-established, as individuals with lower socioeconomic status (SES; i.e., lower levels of education, income) are more likely to experience chronic disease morbidity and early mortality compared to their higher SES counterparts (Adler et al., 1994; Adler and Newman, 2002; Everson et al., 2002; Shishehbor et al., 2006; Byers et al., 2008; Clark et al., 2009; Clegg et al., 2009; Dixon et al., 2019). These stark health disparities are due in part to the fact that lower SES individuals are less likely to adopt preventive health behaviors, such as eating a healthy diet, engaging in regular physical activity, and utilizing preventative healthcare services (Pampel et al., 2010; Stringhini et al., 2010; Nandi et al., 2014). Preventive behaviors are less common among lower SES individuals for numerous reasons, including a lack of resources, inequitable living conditions, and environmental stressors, among many others (Adler and Newman, 2002). Growing evidence also indicates that *communication inequality*, or disparities in the dissemination and use of health information across social groups, may also contribute to the link between SES and health outcomes (Viswanath and Emmons, 2006; Kreps, 2008). Indeed, current messaging campaigns promoting health behavior change are known to be less effective among lower SES individuals, and may actually widen health disparities (Stephens et al., 2012; Lorenc et al., 2013). This is in part because current messaging campaigns fail to consider the sociocultural context that influences receptivity to health messaging (Stephens et al., 2012). Thus, there is a critical need to develop more effective health behavior change messaging campaigns that will specifically resonate with lower SES communities in an effort to promote health equity.

### A Conceptual Framework for Reducing Communication Inequity by Harnessing Neuroimaging

**Figure 1:**
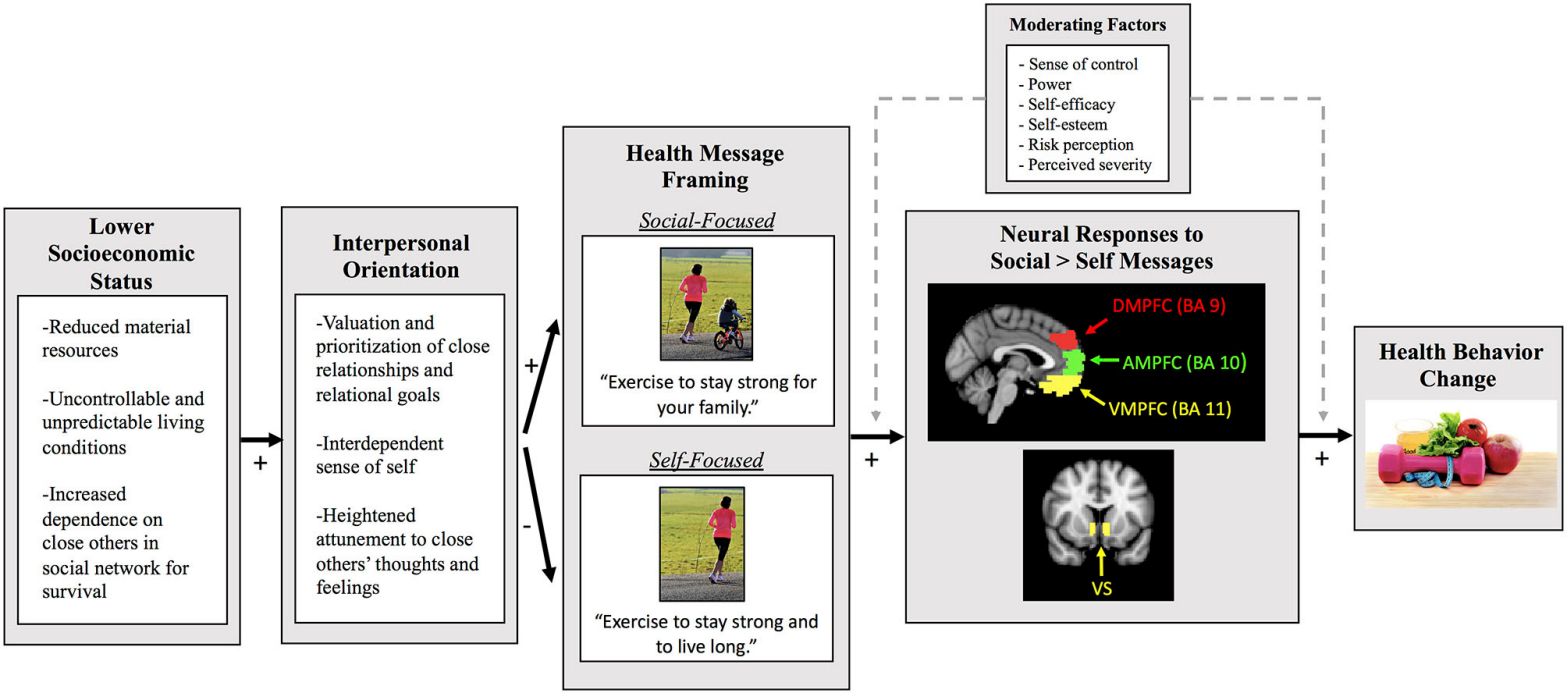
The key tenets of our conceptual framework for improving health messaging for lower SES people. This path diagram illustrates our framework and predictions regarding how the content of health messages may influence neural activity specifically among lower SES individuals. We first highlight that lower objective SES individuals are often constrained by reduced material resources, live in an uncontrollable and unpredictable environment, and depend on close others in their social network for their survival (Markus and Kitayama, 2010; Kraus et al., 2012; Carey and Markus, 2017; Rucker et al., 2018). These conditions associated with lower SES can then give rise to an interpersonal orientation. Specifically, lower SES individuals tend to value and prioritize close relationships and relational goals, express an interdependent sense of self, and have a heightened attunement to close others' thoughts and feelings. We hypothesize that this interpersonal orientation impacts how health information is processed at the neural level, depending upon the content of the health messages. Specifically, we predict that social-focused health messages that highlight how health behavior change can benefit close others will elicit greater activity in the ventral striatum (VS), ventromedial pre-frontal cortex (VMPFC), anteromedial pre-frontal cortex (AMPFC), and dorsomedial pre-frontal cortex (DMPFC), relative to self-focused health messages that highlight how behavior change can benefit the self. This greater neural activity in these key brain regions in response to the social (vs. self) health messages will then predict greater future behavior change. We also include moderating factors that may impact how lower SES individuals process health messages and the extent to which neural activity is predictive of behavior change. Brain images were adapted from Figure 1A in Lieberman et al. (2019).

In the present paper, we propose a novel conceptual framework designed to improve health messaging effectiveness for lower SES individuals. The key tenets of the framework are presented in Figure 1. We argue that features of a lower SES context shape what individuals value, how they view themselves in their social environment, and their attunement to others within their social network. Compared to their higher SES counterparts, lower SES individuals tend to value and prioritize close relationships and relational goals (vs. personal goals), see themselves as interdependent with close others (vs. autonomous), and have a heightened sensitivity to what close others are thinking and feeling (Kraus et al., 2010, 2012; Markus and Kitayama, 2010; Carey and Markus, 2017; Rucker et al., 2018). We hypothesize that this interpersonal orientation impacts how health information is processed at the level of the brain and used to guide behavior. We argue that activity in the ventral striatum (VS) and three subdivisions of the medial pre-frontal cortex (MPFC), the ventromedial pre-frontal cortex (VMPFC), anteromedial pre-frontal cortex (AMPFC), and dorsomedial pre-frontal cortex (DMPFC) will be differentially activated among individuals from lower SES backgrounds, depending upon the content of the health message (Falk and Scholz, 2018). Specifically, we hypothesize that lower SES individuals will show greater activity in the VS and the three MPFC subdivisions in response to “social-focused” health messages that emphasize the social value and benefits to close others of engaging in health behavior change, relative to standard “self-focused” health messages that emphasize the value and benefits to the self. Further, given that activity in the VS, VMPFC, AMPFC, and DMPFC has been shown to predict message-induced behavior change (Falk and Scholz, 2018), we argue that social-focused health messages (relative to self-focused health messages) will induce greater behavior change among lower SES individuals in part because such messages are associated with greater activity in VS and subdivisions of MPFC.

What follows is a review of the cultural/social psychology, communication neuroscience, and social neuroscience literatures that give rise to the conceptual framework outlined above and in Figure 1. We focus our review on empirically-established neural predictors of health messaging-induced behavior change, and how lower SES and/or an interpersonal orientation modulates activity in these brain regions. We next propose a research agenda to test the conceptual framework and discuss ideas for future work. In pulling together these previously-disparate lines of work into a novel conceptual framework, we hope to facilitate efforts to design and empirically test targeted messaging campaigns to close the current gaps in chronic disease disparities across the SES gradient.

### Framing Health Messages to Address the Concerns of Lower SES People

A key starting point of our framework builds on complementary theories and empirical evidence showing that, in the United States, SES shapes how individuals think and feel about the self and others, and how they navigate through their social environment (Kraus et al., 2010, 2012; Markus and Kitayama, 2010; Rucker et al., 2018). Previous work in this area has often operationalized SES based on a combination of material or objective resources, including wealth, income, educational attainment, and occupational prestige (Drentea, 2000; Oakes and Rossi, 2003; Snibbe and Markus, 2005). Other work emphasizes the subjective experience of socioeconomic status, or one's perceived social rank based on the quality and quantity of their material resources relative to others in society (Adler et al., 2000; Cohen et al., 2008; Kraus et al., 2012). Objective measures of SES are commonly used in health neuroscience and health disparities research, and are associated with mental and physical health outcomes (Everson et al., 2002; Lorant et al., 2003; Shishehbor et al., 2006; Clark et al., 2009; Agerbo et al., 2015). Various indicators of objective SES may also differentially predict health behaviors. For instance, beyond access to economic benefits, educational attainment may uniquely impact health-related knowledge, health literacy, and problem-solving skills (Reynolds and Ross, 1998; Braveman et al., 2005). Studies specifically focused on lower SES populations often operationalize “low SES” based on cut-off points, such as having <4-year college degree, having a household income in the lowest tertile/quartile or below the federal poverty line, or working a blue collar or service job (vs. executive or professional; Adler et al., 2000; Everson et al., 2002; Stephens et al., 2007; Stringhini et al., 2010; Kraus et al., 2012).

In addition to individual objective SES, recent work has shown that characteristics of a person's environment, such as neighborhood-level SES (e.g., neighborhood income and education), as well as relative deprivation and economic inequality (at the local and macro level) can predict health outcomes (Kawachi and Kennedy, 1999; Robert, 1999; Diez Roux, 2002; Sampson et al., 2002; Wilkinson and Pickett, 2006; Payne et al., 2017). The economic environment may be just as important to measure as individual-level SES, as it can also shape opportunities for engaging in healthy behaviors. For instance, individuals with higher personal income may live in a food desert where their access to nutritional foods is quite limited. Conversely, individuals with lower personal income may live in a neighborhood surrounded by many parks and recreational areas where they can engage in adequate physical activity. Thus, studying multiple levels of objective SES (i.e., both individual and neighborhood/environmental) can help to capture a more holistic picture of how SES disparities in health behaviors arise, and the messages that should be targeted to each community.

Though objective measures of SES are useful to include in future health messaging studies, subjective SES (e.g., relative rank) has also been shown to predict health outcomes, even above and beyond objective measures of SES (Singh-Manoux et al., 2003, 2005; Demakakos et al., 2008), and is more strongly linked with subjective well-being (Tan et al., 2020). Subjective SES is commonly measured using the MacArthur Subjective Social Status Scale, which asks individuals to place themselves on a 10-rung social ladder based on their income, education and occupation, relative to others in society (Adler et al., 2000). Measuring subjective SES may be particularly beneficial as it can be used as a composite measure (Segal et al., 1970; Webster and Driskell, 1978) that likely represents a cognitive average of different SES indicators (Singh-Manoux et al., 2003). It could also help to identify components of social capital that objective measures miss, such as the prestige of a university that one obtained their degree from or a company that they work for. In sum, SES is a multifaceted construct that can be measured in multiple ways, and should be carefully considered in future fMRI studies. Furthermore, prospective imaging work should consider how different indicators of SES (e.g., objective vs. subjective) could differentially influence health-related brain functioning (Muscatell, 2018).

While objective and subjective SES may have unique effects on the brain and health, previous literature in social psychology posits that lower levels of both components of SES give rise to distinct beliefs, values, and behaviors (Kraus et al., 2010, 2012; Markus and Kitayama, 2010; Rucker et al., 2018). Specifically, lower objective SES individuals are often constrained by reduced material resources, and have fewer opportunities for choice, control, or influence over their outcomes (Kraus et al., 2012). In order to adapt and survive within a constrained and unpredictable environment, lower SES individuals must often rely on their social networks, which tend to be small, dense, and homogenous, made-up of strong and long-lasting relationships (Carey and Markus, 2017). As these critical ties provide support and facilitate survival, lower SES individuals must work to maintain them by prioritizing and helping close others, building trust, and developing a heightened attunement to others' thoughts, feelings and motivations (Putnam, 2000; Curley, 2009; Offer et al., 2010; Carey and Markus, 2017). This interpersonal orientation can also foster an interdependent sense of self, such that group membership and relational roles becomes a core part of an individual's identity (Adams et al., 2004; Oishi et al., 2007; Talhelm and Oishi, 2014; Carey and Markus, 2017). Integrating close others into the sense of self can shift one's own goals, interests, and preferences such that group goals become prioritized over personal goals. In line with this, lower SES individuals have been shown to focus more on group goals that can benefit those in their tight-knit circle, rather than focusing on unique traits, personal motivations and personal goals (Kashima et al., 1995; Carey and Markus, 2017). Further, when navigating through the social environment, people from lower SES backgrounds often use self-expression to blend in with others (Hart and Edelstein, 1992; Weininger and Lareau, 2009) make communal decisions and feel more positive about making the same choice as others (Stephens et al., 2007, 2011), are better at reading others' emotions (Kraus et al., 2010), and engage in more prosocial behavior (Piff et al., 2010), relative to their higher SES counterparts. Thus, a lower SES environment can give rise to a unique set of values and beliefs about the self and others that are context-sensitive, such that lower SES individuals tend to focus on the self in relation to close others and emphasize group goals over personal achievement.

Extending these observations to the health domain, we propose that the tendency of lower SES individuals to adopt a stronger interpersonal orientation, focused on close others over the self, may also influence how health and illness are conceptualized. Specifically, lower SES individuals who are more interdependent and interpersonally oriented may be motivated to maintain their health to fulfill social obligations and avoid being a burden on close others. This pattern has been shown in prior work, such that a more interdependent sense of self predicts the extent to which individuals are concerned that their health problems may negatively impact people close to them (Uskul and Hynie, 2007). Further, interdependence influences how much people experience social-related emotions (e.g., shame and embarrassment) in response to threats to their physical health (Uskul and Hynie, 2014). These interpersonally-focused health concerns may in turn influence whether health messages emphasizing different reasons for engaging in healthy behaviors are attended to and remembered, and whether they are likely to induce subsequent behavior change among a lower SES population (Viswanath and Emmons, 2006). We thus suggest that future health messaging campaigns that aim to target lower SES individuals should consider how health information is framed and contextualized.

So how can we frame health messages to be more persuasive and effective among lower SES individuals? Interestingly, marketers outside of the health domain may have already identified one possible answer. In a study investigating the advertising strategies employed in different socioeconomic contexts (e.g., working-class vs. middle-class), researchers found that working-class ads promoted messages of interdependence, whereas middle-class ads promoted messages focused on differentiating the self from others (Stephens et al., 2007). More specifically, ads targeting working class consumers were significantly more likely to feature images with people, and to focus on interpersonal relationships (e.g., “Take family time further”) and connecting with others (e.g., “Combines the things you love”). Conversely, ads targeting middle-class consumers emphasized the importance of being unique (e.g., “Only one of its kind in the world”) and deviating from the norm (e.g., “Outrun the fashion police”). Thus, while marketers are already making use of the specific values that appeal to different SES groups to sell products, to our knowledge, these messaging strategies have yet to be applied systematically to the health domain.

Current mainstream health messaging campaigns promoting a healthy lifestyle tend to emphasize enhancing personal attributes (e.g., feeling and looking great), maintaining freedom and independence, and reaching personal goals. This self-focused framing may not be effective among lower SES individuals, who tend to be more focused on the well-being and stability of their social network rather than focusing on personal goals and independence. As such, shifting health messages to be more interpersonally-focused may be more congruent with and instrumental for lower SES individuals. Indeed, past cross-cultural work has already demonstrated how tailoring health messages to match individuals' interpersonal orientations can enhance their effectiveness through shifting perceptions of persuasiveness and even improving health behaviors (Kalichman and Coley, 1995; Herek et al., 1998; Kreuter and McClure, 2004; Uskul, 2004; Kreuter et al., 2005; Uskul and Oyserman, 2010). Given this promising work, we suggest that this message framing strategy could be extended to lower SES populations in order to address SES disparities in health. Specifically, we predict that health messages highlighting how health behavior change can positively impact close others (e.g., staying healthy to be there for loved ones) will be more effective among lower SES individuals with an interdependent and interpersonal orientation, compared to health messages focusing on how health behavior change could benefit the self (e.g., reaching personal goals). Integrating the unique values and beliefs about the self and close others observed among lower SES individuals could facilitate the design of messaging campaigns that are more equitable, relevant, and more likely to give rise to behavior change.

## A “Brain-as-Predictor” Approach to Facilitate Message-Induced Behavior Change

Our framework additionally emphasizes that tools from neuroimaging should be harnessed to evaluate message effectiveness and to reveal *why* certain messages are particularly potent predictors of behavior change among lower SES individuals. We believe that a “brain-as-predictor” approach that uses neuroimaging techniques, such as fMRI, both to index neural responses to a task and predict behavior outside of the laboratory, will generate useful insights (Berkman and Falk, 2013). These neuroimaging techniques will be particularly powerful in helping create more equitable health communications when they are coupled with behavioral studies that can enroll larger groups of participants and include individuals from areas not often represented in neuroscience research (e.g., rural areas). Indeed, previous neuroscientific work has successfully used fMRI data to predict health message effectiveness at the individual and population level (Falk et al., 2010, 2011, 2012, 2016; Chua et al., 2011; Wang et al., 2013). Critically, brain activity in response to health messages can predict behavioral changes that are not evident from self-reports alone, and can even be a better predictor of individual behavior change and the future success of public health campaigns (Falk et al., 2010, 2011, 2012). Thus, fMRI data can predict message-induced behavior change above-and-beyond self-reports, perhaps in part because these data provide real-time information about message persuasiveness that subjects may be unwilling or unable to report. Brain imaging can also reveal *why* a particular health message is effective, as it allows for investigation of the neural mechanisms and their associated cognitive processes (i.e., valuation, self-related processing, and social cognition) that facilitate behavior change. Given its advantages, we suggest that neuroimaging techniques need to be harnessed to provide a more robust indicator of message persuasiveness and likelihood of the message leading to future behavior change among lower SES individuals. However, given the expense and difficulty of acquiring fMRI data, following initial “neural focus groups,” knowledge gained from brain imaging research can be extended into follow-up behavioral studies and broader dissemination efforts. In other words, neuroimaging research is foundational for and complementary to behavioral work on message effectiveness (Falk et al., 2012).

Thus far, we have laid out the core tenants of our conceptual framework and argued that neuroimaging research is critical to empirically test the framework and design maximally-effective health behavior change messages. In the subsequent sections, we outline specific predictions from our framework and ideas for future research that could utilize a brain-as-predictor approach to evaluate messaging effectiveness in an effort to improve health behaviors among lower SES individuals. We focus our predictions on the VS and on three subdivisions of MPFC (i.e., VMPFC, AMPFC, and DMPFC) and their hypothesized neurocognitive functions, given that activity in these regions in response to health messages has been shown to predict behavior change (Falk and Scholz, 2018; Lieberman et al., 2019).

### VMPFC/VS and Valuation

The first neurocognitive system that is relevant for understanding message-induced behavior change is that involved in valuation (Bartra et al., 2013; Falk and Scholz, 2018; Hall et al., 2018). A large body of work in affective neuroscience demonstrates that neural regions that make up the “value system,” including the ventromedial pre-frontal cortex (VMPFC) and the ventral striatum (VS), activate in response to a variety of valued stimuli (Sescousse et al., 2013), such as monetary rewards (Levy and Glimcher, 2011; Bartra et al., 2013; Wu et al., 2014), appetizing food cues (Levy and Glimcher, 2011; van der Laan et al., 2011), images of cigarette smoking among smokers (David et al., 2005), and social rewards (Inagaki and Eisenberger, 2013; Inagaki et al., 2016). Literature from communication neuroscience suggests that this system also facilitates value-based decision making, which may be an integral pathway through which thoughts can transform into actions, as people weigh the costs and benefits to calculate the value of their potential choices (Levy and Glimcher, 2012; Falk and Scholz, 2018; DeStasio et al., 2019). More specifically, information about various choices feeds into the VMPFC and VS, which then create a “common value signal” that can be used to contextualize and compare different options within the environment (Chib et al., 2009; McNamee et al., 2013; Falk and Scholz, 2018). Multiple neuroimaging studies have consistently shown that activity in the VMPFC and VS, as well as greater connectivity between these regions (Cooper et al., 2017), during exposure to health messages about smoking (Falk et al., 2011), physical activity (Falk et al., 2015), and sunscreen use (Falk et al., 2010) is associated with increased future behavior change and large-scale information sharing (Scholz et al., 2017) both at the individual and population level (Falk et al., 2012, 2016). Additionally, health messages that explicitly highlight the value of a behavior to the self have been shown to elicit VMPFC activity, and this message-induced activity is associated with downstream behavior change (Vezich et al., 2017).

Given that the value system (e.g., VMPFC and VS) may be tracking how valuable health information is to an individual and predicts subsequent behavior change, we propose that messages that focus on the personal vs. social value of adopting a healthier lifestyle will be associated with differential activity in this system depending on SES. We hypothesize that social-focused health messages, emphasizing the social value and benefits of engaging in chronic disease prevention behaviors for close others, will be associated with greater activity in the value system and future behavior change among lower SES individuals, given their tendency to value others. In contrast, we predict that self-focused messages highlighting the benefits of behavior change for the self will be associated with less activity in the value system and subsequent behavior change among lower SES individuals, given their tendency to place less emphasis on the self.

So far as we are aware, no research has yet explored how the content of messages (i.e., social vs. self-focused) influences activity in value-related neural regions among lower SES individuals. However, we will discuss the social and cultural neuroscience literature that inspires the conceptual framework and hypotheses outlined above. While a handful of studies have investigated how lower relative social standing in more local hierarchies (e.g., within a social network, in a game) modulates activity in the value system during the processing of social cues (Zink et al., 2008; Zerubavel et al., 2015), to date, only one known study has explored socioeconomic influences on neural activation involved in the valuation of social information. Results from this study revealed that lower SES individuals showed greater VS activity while processing information about lower-status individuals (vs. higher-status individuals; Ly et al., 2011). Complementary to the behavioral work discussed in the prior section (Carey and Markus, 2017), these findings further support the idea that lower SES people attribute value to other lower SES individuals, who are more likely to be a part of their social network. Additionally, emerging evidence demonstrates that among individuals from lower SES backgrounds, increased VMPFC and VS activity during social exclusion is associated with greater future conformity to safe driving norms expressed by peers (Cascio et al., 2017). This recruitment of the value system during exclusion may help to regulate negative affect or could indicate a sensitivity to the possibility of social connection, thus susceptibility to social influence.

Although existing literature investigating SES differences in activity within the value system is quite sparse, research in cultural neuroscience that explores neural responses between individualistic and interdependent cultural orientations is informative. For instance, Varnum et al. (2014) demonstrated that priming an interdependent self-construal leads to a decrease in differentiated VS activation in response to monetary rewards for the self or a friend, whereas priming an individualistic self-construal leads to greater VS activity in response to rewards just for the self. This suggests that value-based decision making among people from interdependent cultures (possibly including those with lower SES) may involve the integration of information about how the outcome could benefit friends and family. In contrast, individualistic cultures (possibly including those with higher SES) may primarily focus on personal benefits during value-based decision making.

Altogether, this small collection of neuroscientific work reveals sociocultural differences in neural activation within the value system (e.g., VMPFC and VS), and provides insight into ways to create health messages that are uniquely valuable among lower SES populations. Future imaging work should design health messages that highlight the positive outcomes of behavior change for either the self (self-focused) or close others (social-focused) to evaluate the extent to which they are differentially associated with activity in the value system and behavior change for lower SES individuals. Given that lower SES individuals tend to value the thoughts and feelings of others (Kraus et al., 2012), we predict that health messages that focus on the social value of behavior change (e.g., “Eat healthy food to be a role model for your family”) will be associated with greater activation in the value system and greater behavior change among lower SES individuals, relative to health messages that focus on value to the self (e.g., “Eat healthy food to be your best self”).

### AMPFC and Self-Relevance

Another neural region integral in predicting message-induced behavior change is the anteromedial pre-frontal cortex (AMPFC; Falk and Scholz, 2018). This region is involved in processing information relevant to self-concept, self-esteem, and self-enhancement (Lieberman et al., 2019), and may also play a role in evaluating if decisions are in line with personal beliefs and attitudes (Brosch et al., 2012). One prior study found that AMPFC activation is uniquely associated with processing core personal values (e.g., fighting injustice), as opposed to processing activities that are less related to values (e.g., playing tennis; Brosch et al., 2012). Communication neuroscientists have extended this investigation into the health domain, and have linked activation in the AMPFC in response to health messages with future behavior change (Cooper et al., 2015), as well as the likelihood of sharing the information with others (Baek et al., 2017). Additionally, personally-tailored health messages also activate the AMPFC, and the extent of this activity predicts downstream behavior change (Chua et al., 2011).

Currently, there are no known neuroimaging studies that explore how SES influences activity in the AMPFC in response to health messages; however, cultural neuroscience work has demonstrated cultural group differences in AMPFC activity during self-related processing (Han et al., 2013). For instance, individuals from non-Western countries with an interdependent cultural orientation recruit the AMPFC to represent both the self and a close other (Zhu et al., 2007), and while processing contextual self-descriptions (e.g., “when talking to my mother, I am…”; Chiao et al., 2009). On the other hand, Western individuals with an individualistic cultural orientation show greater AMPFC activity when thinking about the self and not when thinking about others (Zhu et al., 2007; Chiao et al., 2009; Ma et al., 2014). This literature presents neural evidence supporting the classically-studied differences in self-construal between individualistic and interdependent cultures (Markus and Kitayama, 1991), and may provide insight into how lower SES influences AMPFC activity during self-referential processing.

Based on fMRI work focused on neural representations of the self across cultures, we propose that the most effective health messages for lower SES populations will be those that specifically contextualize the self in relation to others. We predict that, among lower SES individuals, health messages emphasizing personal behavior change through its implications for friends and family (e.g., “Exercise to stay strong for your family”) will be associated with stronger activation in the AMPFC, thus inducing future behavior change. In contrast, we predict that health messages focusing on benefits to the self (e.g., “Exercise to stay strong and live long”) will elicit less activity in the AMPFC, producing less downstream behavior change.

### DMPFC and Social Relevance

One last neural region that is important in understanding message-induced health behavior change is the DMPFC, which is involved in a variety of social cognitive processes, such as perceiving and understanding the thoughts, feelings, and motivations of others (Falk and Scholz, 2018; Lieberman et al., 2019). This region is a core node of a broader network of regions, termed the “mentalizing network” (e.g., DMPFC, MPFC, PCC, TPJ, and pSTS), that is consistently activated during social cognitive tasks that involve understanding others' mental states (Frith and Frith, 2006; Mitchell, 2009; Lieberman, 2010). These social cognitive processes may play a central role in message-induced health behavior change, as people may evaluate the impact of their own health behaviors on others, and consider how others perceive them based on their habits and lifestyle. Communication neuroscientists have found activity in DMPFC to be predictive of future behavior change (Wang et al., 2013) and likelihood of sharing health-related information (Baek et al., 2017). Interestingly, some of the central arguments featured in common health messages focus on the social relevance of health behavior change (e.g., smoking is aversive to others; second-hand smoke will harm your baby or child; Wang et al., 2013), suggesting that considering the impact one's behavior has on others may be associated with greater DMPFC activity. These findings further establish the neural underpinnings of processing health information, and reveal how social others can be integrated into the process.

Although no known neuroimaging studies to date have specifically investigated how features of health messages influence DMPFC activity as a function of SES, social neuroscience work has shown that SES modulates DMPFC activity in response to social information. For instance, individuals from lower SES backgrounds show greater activity in the DMPFC while processing social information, angry faces, and negative feedback from others (Muscatell et al., 2012, 2016). Supporting evidence from cultural neuroscience literature has also revealed that East Asians, who tend to be more interdependent, show greater DMPFC activity during social-cognitive processes compared to Westerners, who tend to be more individualistic (Markus and Kitayama, 1991; Han and Ma, 2014). In summary, this suggests that SES, along with levels of interdependence/individualism, modulates neural activation involved in social cognition.

Given that message-induced activity in the DMPFC predicts future behavior change, and that lower SES is associated with greater activity in this region during social-cognitive processes, we propose that socially-relevant health messages will be more effective at inducing behavior change among lower SES populations. We predict that health messages that focus on the social relevance of positive health behavior change (e.g., “Exercise today to be there for your loved ones tomorrow”) will be associated with greater activation in the DMPFC and greater behavior change among lower SES individuals, relative to health messages that focus on just benefits to the self (e.g., “Exercise today to look and feel your best tomorrow”).

### A Research Agenda to Test the Conceptual Framework and Other Future Directions

Based on the foregoing discussion, we ultimately predict that among lower SES individuals, social-focused health messages will elicit greater activation in the VS and sub regions of the MPFC (e.g., VMPFC, AMPFC, and DMPFC), relative to self-focused health messages. Further, we predict that greater activation in these regions in response to the social-focused health messages will predict greater subsequent behavior change. To test these hypotheses, we suggest that future fMRI studies design and test a variety of message framing strategies to determine their effectiveness among lower SES participants. In keeping with prior conceptualizations of “low SES,” this would involve recruiting participants with less than a college degree, those living at or below the federal poverty line, and/or those working blue collar and/or service industry jobs. During the fMRI scan, participants can be randomly assigned to view health messages that focus either on how engaging in a variety of healthy behaviors (e.g., eating a healthy diet, exercising, applying sunscreen, attending screenings) can benefit close others, or how they benefit the self. For example, a social-focused health message promoting a healthy diet could state, “Depend on a healthy diet, so your family can depend on you,” whereas a self-focused message could say, “Depend on a healthy diet to feel and look your best.” Similarly, a social-focused health message promoting cancer screenings could state, “Get regularly screened for cancer so you can grow old with your partner,” whereas a self-focused message could say “Get regularly screened for cancer to live a long and successful life.” In addition to altering the text as outlined above, health messages could also feature different images that are either social-focused (e.g., a family engaging in healthy behaviors together) or self-focused (i.e., a solo individual engaging in healthy behaviors). Message-induced neural activity within the VS and sub regions of the MPFC can then be compared between the two different assigned groups (e.g., social-focused vs. self-focused), relative to a control condition (e.g., “Get regularly screened for cancer”), to see which message framing type elicits the greatest activity in these regions of interest. To assess downstream behavior change, participants can complete follow-up behavior change assessments a few months following the fMRI scan. Further analyses can then test the extent to which message-induced activity in the regions of interest predict future behavior change, depending on the content of the message. Collecting participants' perceptions of the persuasiveness of health messages may also enable researchers to test whether self-reported measures or neural responses to the messages are better predictors of behavior change.

Once these initial studies focused on lower SES individuals are carried out, it will be important for subsequent studies to recruit people across the entire SES gradient and examine similar processes. Comparing the effectiveness of different message framing strategies (and the underlying neural activity) among lower SES people vs. higher SES people will ensure that the effects are specific to lower SES. We predict that compared to higher SES individuals, lower SES individuals will show greater neural activity in the VS and sub regions of the MPFC in response to social-focused health messages (relative to self-focused messages). Conversely, we predict that compared to lower SES individuals, higher SES individuals will show greater neural activity in these regions in response to self-focused health messages (relative to social-focused messages). Further, greater activation in these regions of interest will predict downstream behavior change. Thus, social-focused health messages will be more effective for lower SES people, and self-focused health messages will be more effective among higher SES people.

Beyond our main predictions and example paradigm, there are a myriad of other exciting directions that future imaging studies could take, such as testing the effectiveness of multiple types of social-focused health messages. For instance, in our examples above, we primarily focus on close others, such as interpersonal relationships, given the past literature in social psychology and sociology showing that lower SES individuals tend to have tight-knit social networks (Carey and Markus, 2017). However, it would also be interesting to vary the “social distance” of others featured in social-focused health messages to examine their relative effectiveness. As such, future imaging studies could measure VS and MPFC activity in response to health messages that either emphasize the benefits of an individual's health behaviors to their close family/friends (e.g., sharing a healthy meal together), neighborhood/surrounding community (e.g., creating a shared garden), or even at the national/global level (e.g., environmental impacts of a meat- vs. plant-based diet), and examine if activity in response to a particular type of social message best predicts downstream behavior change.

In addition to varying the social distance of others, prospective fMRI studies could explicitly mention various social roles and identities of the individual, and how maintaining their health could help fulfill their social obligations. Indeed, cultural neuroscience work has shown that making self-judgments about one's social attributes (e.g., social roles/identities) evokes greater activity in a region included in the mentalizing network (i.e., TPJ) and the AMPFC among more interdependent individuals (Chiao et al., 2009; Ma et al., 2014). As such, designing health messages that highlight the link between health behaviors and social identities may also recruit the DMPFC and AMPFC to a greater degree among people from lower SES backgrounds, and could thus elicit increases in future behavior change. For example, emphasizing social roles (e.g., head of household, family caregiver, role model) within the family or community may signal the duties and responsibilities that come along with being a parent, partner, son/daughter, friend, neighbor, and exemplar (e.g., “As the head of your household, staying healthy will help you to continue to provide for your family;” “As your family's caregiver, your health and well-being is critical for maintaining *theirs*;” “As a teacher, be a healthy role model in your students' lives.”). Although focusing on specific social roles and identities could be effective, it is important to note that social identity can influence beliefs and attitudes about health behaviors and could even undermine motivations to change. For instance, lower SES Americans are more likely to view unhealthy behaviors as part of their in-group identity, and making their socioeconomic group membership salient influences fatalistic beliefs about improving health (Oyserman et al., 2007). Future imaging studies could thus include health messages that focus on other aspects of social identity associated with social relations and group commitment, and explore whether this framing elicits more DMPFC and AMPFC activity.

In addition to focusing on how healthy behaviors can facilitate social obligations and foster relationships, future work could design and test messages that emphasize how *unhealthy* behaviors associated with chronic diseases can have relational consequences. For example, social-focused messages that emphasize how chronic illness could harm the quality of relationships or the stability of a social network may induce activation within the DMPFC and AMPFC, and influence behavior change. Additionally, previous findings from an ERP study suggest that lower SES individuals may be more attuned to others in pain, as shown through heightened activity in an ERP component involved in social cognition (Varnum et al., 2015). Future fMRI studies could thus additionally test whether health messages that feature images of loved ones feeling pain and sadness if the individual falls chronically ill induce greater DMPFC activity among lower SES participants.

Lastly, given that group membership and relational roles are an integral part of lower SES individuals' sense of identity (Adams et al., 2004; Oishi et al., 2007; Talhelm and Oishi, 2014; Carey and Markus, 2017), social-focused health messages could highlight how healthy behaviors are in line with ingroup norms. Along these lines, past work has demonstrated that lower SES individuals prefer making the same choice as others (Stephens et al., 2007, 2011). Thus, they might make healthier choices if they are in line with how others in their social network have chosen to lead their lives. Imaging work studying the neural correlates of social influence found that participants who showed greater activity in the DMPFC in response to seeing others' opinions were more likely to shift their judgments to match the opinions of others (Cascio et al., 2015; Welborn et al., 2016). These findings suggest that health messages featuring information about ingroup norms and close others' opinions about health behaviors could induce DMPFC activity, and thus facilitate subsequent behavior change. Additionally, considering that the DMPFC is recruited when individuals are thinking about the mental states of others (Lieberman et al., 2019), health messages could also be framed in a first-person perspective that highlights the thoughts and feelings that close others have toward a certain health behavior (e.g., a partner saying “I think smoking is a disgusting habit.”). Moreover, one imaging study specifically found that lower SES moderated neural sensitivity within the VS and VMPFC to social exclusion or threats to social bonds, impacting susceptibility to social influence (Cascio et al., 2017). Future work could therefore explore if neural activity in response to messages that focus on others' disapproval of unhealthy choices or failing others' expectations predicts future behavior change among lower SES populations.

In summary, there are many opportunities for future work in this emerging field to test the effectiveness of various messaging strategies, which differentially integrate details about the social environment with health information. Further, future studies are needed to explore how neural activity within the VS and sub regions of the MPFC in response to a variety of social-focused health messages predict future behavior change among lower SES individuals. This level of specificity will help to identify the most effective framing strategies that will begin to close the gap in SES health disparities.

## Additional Considerations

While the current framework serves as an overview and presents overarching patterns that are more frequently observed within lower SES populations, it is also important to note that there is likely variability in the expression of an interpersonal orientation among lower SES individuals within the U.S. For instance, Americans (along with Western Europeans) more generally tend to express an independent or individualistic view of the self, compared to individuals from Non-Western countries (Asian, African, Latin-American, southern European), who tend to adopt an interdependent view of the self (Markus and Kitayama, 1991). This country-level individualistic orientation may also influence how health messages are processed, and self-focused messages may be more effective for some lower SES individuals. Future work could additionally explore the intersection of race/ethnicity and lower SES to see whether social-focused vs. self-focused health messages are particularly effective among specific ethnic and racial groups that also emphasize interpersonal relations (e.g., European Americans vs. Asian Americans and Latinx Americans). Other work suggests that there is variability in how interdependence is expressed and valued across different countries. For example, although Americans generally tend to express less interdependence, this may be attributed to a lower valuation of group harmony and duty to the in-group, which are more valued components of interdependence in East Asian countries. Interestingly, relatedness, seeking others' advice, and feeling a sense of group belonging is more prevalent among Americans compared to East Asians (Oyserman et al., 2002), suggesting that the nature of “interdependence” may vary from country to country. This suggests the possibility that, for lower SES Americans, social-focused health messages emphasizing a sense of group belonging/membership (e.g., joining a walking group) could be especially effective. However, future work is needed to test this distinction between feelings of belonging vs. duty to the in-group among lower SES Americans, to examine the specific social-focused health messages that are most effective for this population.

In addition to exploring how message framing influences health behavior change among lower SES individuals, future work should also investigate well-established moderators that could impact how lower SES individuals process health messages and engage in behavior change. For instance, previous work has demonstrated that lower SES individuals have a lower sense of control over their life outcomes, indicated by lower perceived mastery and higher perceived constraints (Lachman and Weaver, 1998; Kraus et al., 2009). This lower sense of control among lower SES groups is also associated with poorer self-rated health; however, lower SES individuals with a higher sense of control tend to rate their health more similarly to their higher SES counterparts (Lachman and Weaver, 1998; Kraus et al., 2009). These findings suggest that a higher sense of control may be particularly protective for lower SES individuals, and could impact the likelihood that individuals engage in healthy behaviors, as they believe that their actions can influence their outcomes. Thus, another possible target for future health messaging campaigns designed to impact lower SES individuals is to address controllability as part of the message. At the very least, perceptions of control should be measured in future work, as a possible moderator of effects.

Another moderator to be considered in future research in this area is social power, often defined as an individual's control over resources and influence over other individuals (Rucker et al., 2018). Past work has shown that low-power individuals express a greater interpersonal orientation, whereas high-power individuals express more of an independent and agentic orientation (Rucker et al., 2018). One may expect that lower SES individuals generally have lower power, but it's important to consider that lower SES individuals can also occupy positions of power within their family (e.g., head of the household or primary income earner) and their communities (e.g., restaurant manager, deacon in a church). The extent to which a lower SES individual holds positions of power within their social environment could thus influence the relevance of social-focused vs. self-focused health messages, as well as whether the individual can engage in and sustain healthy behaviors that could be dependent on members of their household or social network.

In addition to sense of control and power, individual characteristics, such as self-efficacy (Boardman and Robert, 2000) and self-esteem (Twenge and Campbell, 2002) may also impact lower SES individuals' engagement in health behavior change. Specifically, lower SES individuals report lower levels of self-esteem and self-efficacy (Boardman and Robert, 2000; Twenge and Campbell, 2002), which are also associated with risky and unhealthy behaviors and poor mental and physical health (Langer and Rodin, 1976; Rodin et al., 1985; Lachman, 1986; Peterson and Stunkard, 1989; Shannon et al., 1997; Norman et al., 1998; Mann et al., 2004). If an individual doesn't believe that they are capable of achieving their health goals or they negatively evaluate the self, this could undermine initial engagement with health messages or could negatively impact the motivation and desire to engage in behavior change, even if a message is persuasive. Future studies should thus account for these characteristics when specifically exploring health messaging effectiveness among lower SES individuals who may be more susceptible to these negative beliefs and evaluations of the self.

Lastly, perceived risk for getting a disease and the perceived severity of chronic diseases may also shape how health information is processed and applied to behavior within a lower SES population. More specifically, lower SES is associated with lower perceived risk of cancer and less cancer worries (Peretti-Watel et al., 2014; Assari et al., 2019), as well as poor knowledge about the risks of obesity (Pampel et al., 2010). Furthermore, lower SES individuals may also express a low perceived severity of chronic diseases, as they are less exposed to warnings about unhealthy behaviors and tend to see more ads that promote the enjoyment and glamor of these behaviors (Ferrer et al., 2018). Thus, these perceptions of disease risk and severity could impact whether lower SES individuals pay attention to health information and believe that it applies to their own health and well-being.

## Implications and Conclusions: Moving Toward Health Equity

In the present paper, we introduced a novel conceptual framework (Figure 1) that integrates work from social/cultural psychology, communication neuroscience, and social neuroscience to generate testable hypotheses regarding the influence of lower SES on health message-induced neural activity. We argue that using a “brain-as-predictor” approach could shed light on why existing efforts to encourage engagement in a healthier lifestyle are less effective for individuals from lower SES backgrounds. Although there are many possible approaches to encourage lower SES individuals to engage in disease prevention behaviors (e.g., providing individual skills and material resources; Stephens et al., 2012; Lorenc et al., 2013), we argue that making health information more congruent with and relevant for lower SES individuals who tend be more interpersonally oriented will help to encourage behavior change and close the gap in SES health disparities. We predict that among lower SES individuals, health messages highlighting the social implications of engaging in chronic disease prevention behaviors will elicit greater activation in the VS and sub regions of the MPFC (e.g., VMPFC, AMPFC, and DMPFC), relative to self-focused health messages, and that greater activation in these regions in response to the social-focused health messages will predict downstream behavior change.

How can the conceptual framework and research agenda outlined above be utilized to ultimately impact health equity? We see at least two concrete ways in which knowledge gained from this program of research could be rolled-out in public health efforts to improve health behaviors among lower SES individuals. First, based on the results of the studies proposed here, alternative versions of public service announcements promoting healthy behaviors could be created. Those messaging campaigns that have been shown to be more effective in inducing behavior change among lower SES individuals (i.e., the hypothesized social-focused messages) could then be rolled out specifically in lower SES communities. For example, billboards, advertisements at public transit stops, and even TV, radio, and social media public service announcements could be tailored to more effectively speak to and resonate with individuals in the communities where they reside. Second, results from these studies could be used to provide more cultural-tailored training for physicians, nurses, and other health-care professionals so that these providers can more effectively motivate health behavior change across the SES gradient. Along these lines, providers could receive information about how to frame behavior change messages in more socially-oriented terms, and thus more effectively motivate their lower SES patients to engage in behavior change. This could improve patient-provider communication in an effort to achieve health equity. Thus, results from the research agenda outlined here can (and should) ultimately be disseminated to public health and health-care professionals such that the knowledge gained can be implemented in lower SES communities to promote health equity.

In conclusion, there are tremendous opportunities to harness both communication and social neuroscience perspectives to create effective and tailored messages that advance chronic disease prevention efforts among lower SES populations who are more susceptible to poor health outcomes. Our proposed framework serves as a path for future research to address SES disparities in health through improving health messaging effectiveness utilizing a brain-as-predictor approach. Future studies should use brain imaging to understand *which* health messages are most effective in inducing behavior change among lower SES individuals, and *why* such messages are particularly potent. This promising area of work can then be used to design more effective and targeted messaging campaigns that can be carried out at the population-level to help close the current gaps in chronic disease disparities and promote health equity.

## Data Availability Statement

The original contributions generated for the study are included in the article/Supplementary Material, further inquiries can be directed to the corresponding author/s.

## Author Contributions

SB and KM wrote the manuscript. PS and AL participated in the revision and proof-reading process of the manuscript. All authors have approved the final manuscript.

## Conflict of Interest

The authors declare that the research was conducted in the absence of any commercial or financial relationships that could be construed as a potential conflict of interest.
